# Food Safety and Sanitation Implementation Impasse on Adolescents in Kenyan High Schools

**DOI:** 10.3390/ijerph18031304

**Published:** 2021-02-01

**Authors:** Csaba Bálint Illés, Anna Dunay, Charlotte Serrem, Bridget Atubukha, Kevin Serrem

**Affiliations:** 1 Institute of Business Economics, Leadership and Management, Szent István University, 2100 Gödöllő, Hungary; illes.balint.csaba@szie.hu (C.B.I.); kevin.serrem@phd.uni-szie.hu (K.S.); 2 Department of Consumer Sciences, School of Agriculture and Biotechnology, University of Eldoret, Eldoret 1125-30100, Kenya; charlottejes@gmail.com; 3 Faculty of Bioscience Engineering, Katholieke Universitiet Leuven, 3001 Leuven, Belgium; kwamebree@gmail.com

**Keywords:** food handlers, knowledge, sanitation, safety, practice, high schools kitchens

## Abstract

The ability to combat food-borne illnesses in food facilities and institutional catering units require sufficient knowledge on food safety and sanitation standards by food producers and consumers. The aim of the study was to investigate the food safety and sanitation knowledge of food handlers in Kenyan high schools. A cross-sectional study was carried out among 204 food handlers in 50 schools. Questions about knowledge and practice toward food safety and sanitation were asked. Respondents were the most knowledgeable on food contamination (93%), while participants were the least knowledgeable on the importance of protective attire when distributing foods to learners (50%). One-way ANOVA revealed a significant difference between gender and food handlers’ behavior and practice (F = 19.886, ρ = 0.00 < 0.05) as well as between job tenure and practice of food safety and sanitation (F = 17.874, ρ = 0.00 < 0.05). Multiple regression analysis established that knowledge contributed to 44.1% of the behavior and practice of the food handlers. It is concluded that food handlers have a fair knowledge despite lack of training, motivation, and facilities to maintain quality standards. It is recommended that the Kenyan Government develop and implement guidelines through school feeding policy that would ensure that food safety and sanitation practices are implemented and utilized by Kenyan high schools.

## 1. Introduction

Food-borne diseases and outbreaks are crucial contributors to morbidity and mortality worldwide [1,2]. Every year, slightly over one-third of the entire population in developing countries is affected by food-borne diseases [3]. For instance, this is evident in West African countries such as Nigeria and Cameroon, which have had a reputation of not enforcing regulations and public ignorance on food safety, food fraud, and poor knowledge of food safety, awareness, and practice among consumers [4]. Likewise, in Asia, food-borne diseases pose the second greatest risk faced by the Chinese in daily life after earthquakes [5]. According to the European Food Safety Authority [6], approximately 48.7% of food-borne diseases were associated with the catering at both institutional and food service establishments, which should be a sector at the epitome of food hygiene and safety, setting trends for other industries to follow.

Education institutions such as primary and high schools’ school feeding programs play a pivotal role in the fight against hunger and malnutrition. When properly designed, they have the potential to improve diets, nutrition knowledge, and practice for all beneficiaries, including service providers and students [7,8,9]. Emphasis on the implementation of food safety measures in school feeding programs are pegged on the vulnerability of school-going children and adolescents to food-borne diseases due to their weaker immune systems compared to adults [10]. Coupled with inadequate infrastructure and paucity of food safety knowledge, food service providers such as food handlers have caused a surge in food-borne disease outbreaks in schools [11].

A significant role in food contamination has been attributed to human handling error by food handlers who are responsible for numerous outbreaks of food poisoning [12]. A study by Rowell [13] comparing high outbreak and non-outbreak facilities revealed that more than 65% of the meals associated with outbreaks were prepared or handled by employees who were infected, while 35% was a result of bare hand contact on food. According to Basch [11], food worker practices can result in the direct and indirect transmission of pathogens through the contamination of food. Foods have a higher chance of being mishandled during preparation, processing, or storage than during consumption [14]. Some practices that may result in food contamination within food service environments include obtaining food from unsafe sources, the use of contaminated equipment, inadequate cooking of foods, and personal hygiene [15,16]. Additionally, improper holding times or inadequate temperatures during food processing especially for ready to eat foods (RTE), which are prepared in advance before consumption and subject to adverse temperature violations, lead to conditions that promote bacterial growth [17]. Appropriate food-handling practices and adequate personal hygiene minimize the transfer of pathogens from handlers to consumers [15]. According to Rowell [13], training is the best way to combat food safety challenges. As part of regulation, the Food and Drug Administration (FDA) ensures that managers are certified as food protection managers, during which they should be well equipped and knowledgeable with regard to food-borne disease prevention, principles of the Hazard Analysis and Critical Control Points (HACCP), and the requirements of the food code [18].

In developing countries such as Kenya, the public is exposed to food-borne pathogens due to changing lifestyles, globalization, changing microbial ecology, and reduced host immunity, which increases the risk of infection [19]. According to the World Health Organisation/ Food Agricultural Organisation [20], the main food-borne diseases are aflatoxin-poisoning gastroenteritis, cholera, dysentery, and brucellosis. For example, in the last 5 years, Kenya has experienced a surge in reported cases of cholera, both in households and institutions such as hospitals, universities, hotels, and restaurants, resulting in some fatalities [7].

Kenya being a developing and middle income country lacks documented guidelines through a school feeding policy on food safety and sanitation practices for use in schools, especially when administering food to school-going children and adolescents. Kenyan schools seem to provide feeding services to children and adolescents based on acquired knowledge and the availability of funds and resources, jeopardizing the health and well-being of the children and adolescents at a critical time of their lives, where a poor foundation of health will lead to lifetime complications. Therefore, the objective of this study was to evaluate the knowledge and practice of food handlers in Kenyan high schools.

## 2. Materials and Methods

A cross-sectional study was conducted in December 2019 and March 2020 to ascertain the level of knowledge and practice of food safety and sanitationof food handlers in Kenyan high school kitchens, catering for students aged 15 to 18 years. The target population included the catering manager and the cooks who were in charge of the kitchen. The study was conducted in eight counties, namely Nakuru, Uasin Gishu, Nandi, Kakamega, Nairobi, Kisumu, Likipia, and ElgeyoMarakwet, where 50 boarding high schools were selected for the study.

Sample size was determined by using a formula proposed by Mugenda and Mugenda [21].

(1) $$N=\frac{Z^{2}p\left(1-p\right)}{d^{2}}$$

where

*N* = the desired sample size.

*Z* = the z score at the required confidence level = 0.05 (*Z* = 1.96).

*p* = the proportion in the target population estimated to have characteristics being measured.

*d* = permissible marginal error (the level of statistical significance set at 0.005).

Purposive judgmental sampling was used by the researchers to select the 8 counties out of a total of 47 counties in the country, and 50 out of a total of 3000 high schools in Kenya for the study. This was carried out based on preliminary knowledge of the availability of boarding high schools in various counties. A catering manager from each of the 50 high schools was interviewed, and systematic random sampling was used to select particular cooks in various high school kitchens.

The questionnaire was divided into 5 sections. Section A addressed the socio-demographic details of the respondents, Section B addressed food handlers’ training and HACCP awareness, Section C included food safety knowledge, which comprised 3 constructs, transmission of food-borne diseases (with 8 items), personal hygiene (10 items), and food contamination (6 items). The total score for knowledge was 24, and each scored 1 if the answer was right and 0 for a wrong answer or “I don’t know”. Food handlers were considered knowledgeable if they achieved a score of 50% and above. Food handlers’ food safety practices were assessed using 14 items. Participants were asked to score according to the frequency of these practices: 1 = never; 2 = occasionally; 3 = sometimes; 4 = often; 5 = always. A pilot study was conducted on 10 food handlers, which led to a slight restructuring and rewording of the questionnaire. The reliability and validity of various sections of the research instrument was determined at a range of 0.63 to 0.79.

Data analysis was carried out using SPSS software for windows version 23.0. Data were presented in mean and frequencies (%). A one-way ANOVA test was used to establish relationships between demographic variables and knowledge and practice. A *p*-value of less than 0.005 was considered statistically significant. Multiple regression was used to assess the influence of Kenyan high school food handlers’ knowledge in relation to their practice.

Ethical approval was granted by the National Commission for Science, Technology and Innovation, (NACOSTI) in Kenya permit number, NACOS-TI/P/1981086/28440. Participation in the study was voluntarily, and respondents were assured of anonymity and utmost confidentiality of the information provided.

## 3. Results and Discussion

There are two types of schools in Kenya, day schools and boarding schools; it is optional for the day schools to offer food to students, as students will consume the majority of their meals at home. On the other hand, it is mandatory for boarding schools to offer meals to students, as students consume the majority of their meals in schools. Additionally, all boarding schools in Kenya prepare their meals at the various institutions. As a precautionary measure, the authors chose to use boarding schools for the study, as they prepared food in the institutions.

### 3.1. Socio Demographic Characteristics

Table 1 shows the demographic characteristics of the respondents. The study featured 50 catering managers and 154 cooks. Of the 204 respondents, the majority (76%) were men, while women were only 23%. The greatest percentage (81%) of the respondents were aged 36–40 years. Only 47% had attained high school diplomas as their highest form of education. With respect to experience, most of the respondents (60%) had over four years of experience as food handlers, and 59% had never previously worked in the food service industry.

### 3.2. Training on Healthy Food Preparation, Food Safety, and Sanitation

The study sought to establish whether food handlers underwent in-service training on healthy food preparation, food safety, and sanitation. The results in Table 2 show that slightly less than half of the respondents 47.5% (97) confirmed being trained. However, 52.5% (107) of the respondents mentioned they had never undergone any form of training on either healthy food preparation or food safety and sanitation. These findings were in sharp contrast to those of Sibanyoni and Tabit [10], whose study of South African schools revealed that 70% of the food handlers had received in-service training for safe food handling in their National School Nutrition Program (NSNP) food preparation facilities. Similarly, according to Ncube et al. [22], the majority (64%) of the food handlers in Zimbabwe had not received basic food safety training, as food safety training was not mandatory in Zimbabwe as it is in Brazil, Malaysia, and European countries [23]. Proper and adequate training should be given to newly employed food handlers, without which they should not be allowed to handle food [24,25]. Adequate knowledge and experience in food safety is imperative for the effective formulation of food safety programs in food service establishments [26]. Moreover, food safety training should prioritize techniques that advocate for behavioral change and the obtainment of recommended food hygiene skills [27,28].

### 3.3. Types of Trainings

Table 3 shows the food handlers’ responses to the different types of training they have undergone. More than half 56.9% (116) of the respondents received training on personal hygiene. About 51.5% (105) received training on purchase procedures, while a similar number, 50.5% (103), did not receive training on pest control. Furthermore, 52.5% (107) of the food handlers affirmed that they had received training on equipment cleaning procedures, whereas only 58.3% (119) of the food handlers had received training on food safety. A similar study conducted by Sibanyoni and Tabit [10] revealed the significance of an effective school feeding policy, as the South African school food handlers yielded much better results in comparison to the current study. With regard to personal hygiene, purchase procedure, and pest control, food handlers in that study achieved higher scores, 71.7%, 73%, and 63.3%, respectively. Additionally, in equipment cleaning procedure, kitchen operation policy, and food safety, their study yielded 64.8%, 65.5%, and 82.4% respectively. These further stem from the fact that in service, training plays a vital role in improving food handling practices and the attitudes of food handlers [1]. Inadequate training of food handlers has led to a lack of adequate food safety knowledge and food handling skills [29,30].

### 3.4. Knowledge and Utilization of Hazard Analysis and Critical Control Points (HACCP)

Food handlers’ knowledge on HACCP is shown in Table 4. Knowledge of HACCP is of utmost importance, as it is the basis for food safety and sanitation procedures in the kitchen, allowing food handlers to take necessary preventive measures. The study revealed that the majority of the food handlers 83.3% (170) did not have knowledge on HACCP. This was further affirmed, as 85.3% (174) of the food handlers mentioned that HACCP procedures were barely implemented in the various institutions in which they worked. These results resonate closely to those of Sibanyoni and Tabit [10], whose study indicated that 91.4% of the food handlers in South African schools did not know what HACCP was. Additionally, a similar percentage of respondents revealed that most of the institutions they worked for did not have an HACCP program in place. These findings were also similar to those of Ncube et al. [22], where a vast majority (96%) of the food handlers in Zimbabwe neither received training nor practiced HACCP procedure. Furthermore, these results indicate that an overwhelming number of institutions neglect the use of important tools such as HACCP, hence highly pre-exposing learners to the risk of food-borne diseases [31]. HACCP is a requisite in the global food supply chain that minimizes the occurrence of negative effects of food safety to consumers [32,33]. According to de Cunha et al. [27], HACCP training should be provided every 6–12 months and its adequacy evaluated regularly.

### 3.5. Food Sanitation and Safety Knowledge

The frequencies of knowledge scores for the three knowledge constructs (transmission of food-borne diseases, personal hygiene, and cross-contamination) are shown in Table 5. It was observed that food handlers scored highly on questions pertaining to food contamination, with a mean of 80%. Of concern was that nearly half of the respondents (45%) lacked adequate knowledge that food prepared long in advance gave microbes time to grow. In contrast, a study by Thelwell-Reid [34] on the retraining of rural Jamaican food handlers observed that a majority of the population, 76%, had adequate knowledge on the influence of time between the preparation of food and the growth of bacteria. Additionally, Nkhebenyane et al. [35] unveiled that the majority (64%) of South African Hospice food handlers were aware of food poisoning risks involved with the early preparation of meals, while a further 68% were conversant with the danger of reheating food before consumption. A substantial percentage (63%) of respondents disagreed with the fact that the HIV virus could be spread through food. On the contrary, Thelwell-Reid [34] reported that the majority, 89%, of the participants in their study had the knowledge that HIV cannot be spread through the consumption of food.

With regard to personal health and hygiene, 63% of the respondents agreed that food handlers could handle food with injuries such as wounds as long as the wound was covered. This finding was similar to that of Nartey et al. [36], who reported that 67.5% of the catering staff in Ghanaian high schools agreed to the fact that handling food with wounds may be a potential cause for food-borne illnesses. Additionally, other scholars found much higher scores in similar studies conducted on the threats that wounds pose in cooking of food. They include Angelillo et al. [37] (99%), Giritlioglu et al. [38] (86%), and Tokuc et al. [39] (93.2%). In relation to the importance of wearing protective attire while cooking and serving students, the study observed a low percentage (35%) of participants. These findings were similar to those of Auad et al. [40], who after undertaking a similar study on Brazilian food trucks found that 97.7% of the food handlers only wore clean uniforms exclusively during food service to consumers, and none of them barely wore protective clothes such as aprons.

Furthermore, other knowledge items that scored low, 46% and 48%, were wearing gloves while handling food protects food service staff from infection and the importance of wearing a cap or chef’s hat when touching or distributing foods to learners, respectively. These findings differed from observations made by Nartey et al. [36], who found that 95% of the caterers in high school wore protective clothes such as gloves, chef’s hats, and aprons while cooking or during the service of food to learners. Likewise, Giritlioglu et al. [38] and Huang [41] reported similarly higher scores of knowledge among food handlers, 97.6% and 82.9%, respectively. On the other hand, a study by Kariuki and Orago [42] in Embu Municipality in Kenya found lower knowledge scores where 21% of the food handlers did not wear protective clothing while preparing food. Additionally, the protective clothing worn by over 30% of the participants was extremely dirty. According to Nkhebenyane et al. [35], only about 2% of the food handlers in South Africa utilized gloves fully, especially when dealing with unwrapped foods, this is despite their tracing on door contamination. Moreover, the study also established that 63% of respondents disagreed that it was acceptable to wash hands at the kitchen sink after visiting the bathroom or lavatory. These findings were consistent with those from a study by Thelwell-Reid [34] who reported that 93.1% of food handlers signified it was not allowed to wash dirty hands in the kitchen sink, as it would lead to the contamination of utensils or food items.

In relation to food contamination, of concern was a mere 36% of the respondents who were of the view that ready-to-eat food could be prepared on the same cutting boards used to prepare meat. These findings contradict those from a previous study by Santos et al. [43], where there were higher knowledge levels of hand washing after sneezing or nose blowing, which stood at 94%. On the other hand, Nguyen et al. [44] found that almost the entire (98%) food service consumer market in Hanoi was aware and cautious of the dangers of food contamination; hence, it heavily advocated for the separation of kitchen utensils such as tongs and knives used for raw and cooked food, respectively. A majority (93%) of respondents were of the view that washing hands with soap in the bathroom was more hygienic, rather than in the kitchen sink, as it would lead to the contamination of either utensils or food stuff [39].

### 3.6. Demographics in Relation to Knowledge Parameters

Analysis of variance (ANOVA) was conducted to determine the correlation between food handlers’ knowledge and food safety and sanitation (Table 6). In relation to gender, male food handlers were more knowledgeable on food transmission diseases and food contamination with average means of (2.833 and 2.761) and (2.683 and 2.633) for males and females, respectively. These findings were in line with those ofMohd Yusof et al. [45], who found out that male food handlers were more knowledgeable on food poisoning and contamination, after carrying out a similar study in a university in Malaysia. On the contrary, a study conducted by Auad et al. [40] revealed that female Brazilian food truck food handlers were more knowledgeable (8.25) compared to their male counterparts (6.97), while Abdul-Mutalib et al. [46] found no significant correlation between gender, education level, age, and work experience. In relation to age, there was a significant difference between food handlers’ age and all knowledge constructs, which included food transmission diseases (F = 5.188, ρ = 0.001 > 0.05), personal health and hygiene (F = 6.824, ρ = 0.00 < 0.05), and food contamination (F = 4.712, ρ = 0.001 < 0.05) (Table 6). The study revealed that in all instances, knowledge decreased with increase in age. Earlier, Table 2 indicated that food handlers aged 25 to 35years were more knowledgeable on food transmission diseases (mean = 2.930) than those aged 65 and above years of age (mean = 2.375). Similar outcomes were observed for personal health and food contamination knowledge constructs, with means of 2.589 for 46 to 55years of age and 2.4 for 65 years and above. Auad et al. [40] reported contrasting results that food handlers aged 40 and above were the most knowledgeable (8.17) compared to their younger counterparts aged 26–40 (7.14), although the differences were not significant. Similarly, a study conducted by Adetunji et al. [47] on the personal hygiene of food handlers in Saudi Arabia revealed no significant differences in knowledge and age relationships, although the food handlers showed positive knowledge toward personal hygiene. Additionally, after carrying out a similar study on food handlers in Zimbabwe, Ncube et al. [22] found that the older the food handlers were, the less knowledgeable they were toward food safety, but on the other hand, the better the levels of attitude they had toward food safety.

ANOVA yielded statistical significance between the length of time the respondents had worked and their knowledge levels of food safety, food transmission diseases (F = 10.988, ρ = 0.000 < 0.05), personal health (F = 18.082, ρ = 0.000 < 0.05), and food contamination (F = 18.666, ρ = 0.000 < 0.05). The results revealed that in all parameters of knowledge, the longer the respondents worked, the more cautious they became about food safety. Similar findings were reiterated by Ncube et al. [22], who revealed that the majority of the food handlers (72%) and (94%) acquired more food safety knowledge and attitude skills, respectively, the longer they worked in a particular establishment.

### 3.7. Practice of Food Safety by Food Handlers

Food handler behavior and practice are portrayed in Table 7. The study observed a fairly adequate level (mean: 4.008) of food safety practices among food handlers in Kenyan schools. These findings mirror the results of Ko [48], who reported a practice average of 4.03 and 4.29 on food sanitary knowledge and behaviors respectively among university restaurant employees. Only about half of the respondents (2.882) used color-coded chopping boards in the various kitchen facilities in which they worked. This outcome resonates with that of Ncube et al. [22], whereby the majority (64%) of the food handlers were untrained and failed to use color-coded chopping boards in the kitchen, greatly increasing the risk of cross-contamination, especially for uncooked foods. On the other hand, a study conducted by Soares et al. [49] indicated that 79.1% of Brazilian school food handlers, who were familiar with HACCP procedures, as it was mandatory in the various facilities in which they worked, always ensured the use of color-coded chopping boards when preparing food. Furthermore, slightly above one-half (2.907) of the participants wore gloves in case of injury while cooking. The outcome of the study echoed that of Nkhebenyane et al. [35], whereby only 36% and 19% of trained and untrained food handlers respectively of South Africa’s hospice facilities occasionally used gloves. The study mentioned that food handlers mostly wore gloves while touching and distributing unwrapped foods so as to curb the risk of cross-contamination. Additionally, a similar study by Ko [48] revealed that 99.6%of the food handlers (University restaurant employees) wore gloves when handling raw ready-to-eat foods and in case of wounds on their hands. The current study also revealed that slightly above one-half of the respondents used the three-sink method while washing utensils. This was partly because the majority of the institutions that participated in the study were old institutions thatwere not modernized with adequate facilities to allow the use of the three-sink system. In addition, Abdullah [15] reported a similar outcome: slightly above one-half (3.79) of the food handlers in universities in Malaysia used the same method while washing utensils.

### 3.8. Demographics in Relation to Practice

The study indicated a significant difference (F = 19.886, ρ = 0.00 < 0.05) between the gender of food handlers and the behavior or practice toward food safety (Table 8). Male food handlers (4.078) were found to have a high level of practice of food safety in relation to their female counterparts (3.762). Mohd Yusof et al. [45], after carrying out similar studies on Malaysian University food handlers, found no relationship between gender and the practice of food safety in the institution. Other studies by various scholars revealed contradictory results, such as Sibanyoni and Tabit [10] and Nkhebenyane et al. [35], whose studies conducted in South Africa and Zimbabwe, respectively, both indicated that the majority of the female food handlers practiced food safety much more than their male counterparts. This was due to a large number offemale employees having been employees in various school kitchen facilities, as culturally such jobs were referred to as women’s jobs.

The study also highlighted a significant difference (F = 18.119, ρ = 0.00 < 0.05) between respondents’ age and practice of food safety and sanitation, further stating the older category 56–65 years of food handlers most likely (4.278) to practice food safety and sanitation. The length of time spent in a particular job combined with the repetitive tasks performed is the prerequisite of experience in a particular career. Similar sentiments were echoed by Sibanyoni and Tabit [10] after carrying out similar studies on food handlers, who found that there was a high significance between age or work experience of the food handler, implying that the longer the food handlers worked, the more they observed hygienic practices. On the contrary, Mohd Yusof et al. [45] failed to find any significant differences between the ages and work experiences of the Malaysian food handlers with knowledge, attitude, and practice.

The study also realized significant differences (F = 9.543, ρ = 0.00 < 0.05) between higher education levels and practice of food safety and sanitation, as food handlers who attended technical colleges (4.051) best practiced food safety and sanitation. This outcome also echoed that of Mohd Yusof et al. [45], who concurrently carried out research on both food handlers and dietetics students at the University of Malaysia. The study found out that the dietetics students had better knowledge and practice on comparison to the food handlers; this was attributed to the fact that they were better educated and had better education levels. Furthermore, various other scholars are in agreement with this finding. According to Soares et al. [49] and Abdullah [15], there is a common consensus that generally, the majority of the food handlers have lower levels of education, with the majority having finished high school. Despite this, a food handler with any form or level of education has a higher perception of knowledge and practice in comparison to those without any formal education.

### 3.9. Multiple Regression on the Influence of Knowledge Parameters and Practice

The results of Table 9 reveal the influence that the constructs of knowledge have on the practice of food safety and sanitation. The results indicate that the standardized coefficient beta and *p*-value of knowledge on food contamination were positive and significant (beta = 0.152, *p* < 0.05).Therefore, a unit increase in knowledge on food contamination results is an improvement in behavior and practice by 0.152 units. The effect of knowledge on food contamination is shown by the t-test value of 2.768, which implies that the effect of knowledge on food contamination surpasses that of the error. It was also observed that food handlers’ knowledge on food transmission diseases had a coefficient of estimate that was significant based on β4 = 0.526 (*p*-value = 0.000, which is less than α = 0.05). Therefore, units increase in food handlers’ knowledge on food transmission diseases results in an improvement in behavior and practice by 0.526 units. The effect of food handlers’ knowledge on food transmission diseases is shown by the t-test value of 9.367, which implies that the effect of food handlers’ knowledge of food contamination surpasses that of the error. Generally, the findings indicate that the independent variable knowledge (whose constructs included knowledge on personal health, knowledge on food contamination, and knowledge on food transmission diseases) contributed up to 44.1% of the variation in behavior and practice of the food handlers, as explained by R2 of 0.441, which revealed the adequacy of the model as a good prediction. Furthermore, Table 9 shows that the F-value of 39.235 with a *p*-value of 0.00 was significant at 5%, indicating that the overall regression model is significant; hence, the joint contribution of the independent variables was significant in predicting behavior and practice.

## 4. Conclusions

Kenyan’s high school food handlers possess fairly adequate knowledge on food contamination and fairly low knowledge levels on personal health hygiene and the transmission of food-borne diseases. More than half of the study participants have never received any form of training or refresher courses on food safety and sanitation, while almost none of the participants know what Hazard Analysis Critical Control Point (HACCP) is. A majority of the respondents admit that none of the kitchen facilities they work for carry out HACCP procedures, hence compromising the health and safety of high school student by increasing susceptibility to food-borne illnesses.

### Recommendation

Kenya requires documented guidelines as school feeding policy that are sustainable and at the same time all-inclusive. It is paramount that food handlers in Kenyan schools receive regular training sessions and for the professionals to receive regular refresher courses on food safety and sanitation, as it is evident from the study that knowledge of food safety and sanitation has a substantial impact on practice, further implying that an increase in knowledge will most likely increase the level of practice.

Additionally, the Hazard Analysis Critical Control Points (HACCP) should be uniformly implemented across all commercial institutional kitchens so as to ensure high surveillance of food through the various stages the food has go through while processing. Processes include temperature control, cross-contamination, proper cooking of foods, and deterioration (pathogens found in food).

Additionally, the policy could be emulated or incorporated by the wider East Africa member states as they face similar challenges. The authors should discuss the results and how they can be interpreted from the perspective of previous studies and of the working hypotheses. The findings and their implications should be discussed in the broadest context possible. Future research directions may also be highlighted.

## Figures and Tables

**Table 1:** Socio-demographic characteristics of food handlers in Kenyan secondary school kitchens.

		Frequency	Percent
Personnel	Managers	50	24.5
	Cooks	154	75.5
	Total	204	100
Gender	Male	158	77.5
	Female	46	22.5
	Total	204	100
Age	25–35 years	78	38.2
	36–45 years	81	39.7
	46–55 years	28	13.7
	56–55 years	8	3.9
	over 65 years of age	9	4.4
	Total	204	100
Highest educational level	primary school level	93	45.6
	high school level	96	47.1
	college	14	6.9
	bachelors degree	1	0.5
	Total	204	100
Years as food handler	less than one year	15	7.4
	one	34	16.7
	two	16	7.8
	three	16	7.8
	four	123	60.3
	Total	204	100
Work experience in food service	yes	84	41.2
	no	120	58.8
	Total	204	100

**Table 2:** Food handler training on healthy food preparation, food safety, and sanitation.

Provision of Training			Frequency of Training		
	Frequency	Percent		Frequency	Percent
Yes	97	47.5	Once every term	26	12.7
No	107	52.5	Twice every term	22	10.8
			Others	49	24
Total	204	100	Total	97	47.5

**Table 3:** Types of training under taken by food handlers in Kenyan secondary school kitchens.

*n* = 204	Yes/No	Frequency	Percent
Personal hygiene	yes	116	56.9
	no	88	43.1
Purchase procedures	yes	105	51.5
	no	93	45.6
Pest control	yes	101	49.5
	no	103	50.5
Equipment cleaning procedure	yes	107	52.5
	no	97	47.5
Kitchen operations polices	yes	114	55.9
	no	90	44.1
Food safety training	yes	119	58.3
	no	85	41.7
Food allergy procedure	yes	103	50.5
	no	101	49.5
Healthy cooking practices	yes	95	46.6
	no	109	53.4

**Table 4:** Knowledge of Hazard Analysis and Critical Control Points (HACCP) by food handlers in Kenyan secondary school kitchens.

Knowledge and Use of HACCP		Frequency	Percent
Food handlers’ knowledge of HACCP	yes	34	16.7
	no	170	83.3
	Total	204	100
Use of HACCP program in school	yes	30	14.7
	no	174	85.3
	Total	204	100

**Table 5:** The frequency of knowledge scores for food-borne diseases transmission, personal hygiene, and food contamination of food handlers in Kenyan high school kitchens (% and the number of respondents in brackets).

Knowledge Constructs	Transmission of Food-Borne Diseases Statements	Agree %	Disagree %	I Don’t Know %
Transmission of food-borne diseases	Well-cooked foods do not have germs	68 (139)	32 (65)	
	Cholera can be spread through food	77 (156)	24 (48)	
	Healthy people can cause illness by carrying germs to food	67 (137)	33 (67)	
	Vegetables and raw salads may be a media for transmitting harmful microbes	90 (184)	10 (20)	
	The HIV virus can be spread through food	29 (60)	63 (129)	3 (5)
	You can tell if food is unfit for consumption by smell, taste, and look	93 (189)	3 (5)	3 (5)
	Food prepared too long in advance might give microbes time to grow	62 (126)	40 (63)	5 (10)
	Fresh meat always has microbes on the surface	72 (147)	20 (41)	8 (16)
	Food transmission diseases	74	32 (65)	
	Personal health and hygiene statements	Agree %	Disagree%	I don’t know %
Personal health and hygiene	Hands can be washed with water alone before handling raw meat	17 (35)	80 (164)	3 (5)
	You can prepare food with a wound on your hand if the wound is covered with a bandage	63 (128)	37 (76)	
	It is not necessary to wash hands so as to handle food that is already cooked	15 (31)	85 (173)	
	Hands should be properly washed after sneezing or blowing your nose	93 (189)	7 (15)	
	Wearing gloves while handling food protects food service staff from infection	35 (72)	46 (97)	17 (35)
	After using the bathroom, hands can be washed in the kitchen sink	37 (75)	63 (129)	
	You should always change your foot wear when you leave the kitchen and go out	53 (108)	42 (85)	3 (5)
	Do you always check the use-by dates of food before using them?	90 (184)	7 (15)	3 (5)
	Do you wear a cap or chef’s hat when touching or distributing foods to learners?	48 (100)	52 (104)	
	Does the school provide you with an adequate food-handling uniform?	79 (162)	21 (42)	
	Personal health and hygiene	70		
	Food contamination statements	Agree %	Disagree %	I don’t know %
Food contamination	Foods prepared with many steps increases handling and the possibility of food contamination	73 (149)	27 (55)	
	Food preparation surfaces can contaminate food	83 (169)	17 (35)	
	Food-borne diseases can result from storing raw meat and cooked foods in the same refrigerator	82 (168)	18 (36)	
	Ready-to-eat foods can be prepared on the same cutting boards that were used to prepare meat	36 (73)	64 (131)	
	Foods can be contaminated with microbes by coming in to contact with unsafe foods	93 (189)	8 (15)	
	Meat cutting boards, slicers, and knives should always be sterilized after use	85 (174)	15 (30)	
	Food Contamination	80		

**Table 6:** Demographic characteristics and level of food knowledge of food handlers in Kenyan high school kitchens.

Demographics Characteristics		Food Transmission Diseases			Personal Health			Food Contamination		
		Descriptive Statistics	ANOVA		Descriptive Statistics	ANOVA		Descriptive Statistics	ANOVA	
		Mean (SD)	F	Sig.	Mean (SD)	F	Sig.	Mean (SD)	F	Sig.
Gender	Female	2.68(0.47)	4.411	0.037	2.51(0.14)	0.013	0.911	2.76(0.18)	19.773	0.000
	Male	2.83(0.14)			2.51(0.13)			2.63(0.18)		
Age	25–35 years	2.93(0.61)	5.188	0.001	2.47(0.15)	6.824	0.000	2.72(0.77)	4.712	0.001
	36–45 years	2.74(0.24)			2.53(0.13)			2.75(0.19)		
	46–55 years	2.79(0.13)			2.59(0.06)			2.74(0.06)		
	56–65 years	2.63(0.00)			2.40(0.00)			2.50(0.00)		
	Above 65	2.38(0.00)			2.40(0.00)			2.83(0.00)		
Education levels	Primary	2.71(0.20)	2.910	0.036	2.49(0.12)	3.206	0.024	2.74(0.18)	0.884	0.450
	Secondary	2.88(0.55)			2.52(0.16)			2.73(0.16)		
	College	2.89(0.44)			2.59(0.02)			2.77(0.25)		
	Degree	2.50(0.42)			2.60(0.13)			2.50(0.17)		
Experience	Less than one year	2.48(0.14)	10.988	0.000	2.31(0.05)	18.082	0.000	2.61(0.13)	18.666	0.000
	One	3.16(0.87)			2.44(0.10)			2.6(0.14)		
	Two	2.83(0.14)			2.63(0.06)			2.59(0.11)		
	Three	2.86(0.01)			2.53(0.05)			2.85(0.17)		
	Four	2.72(0.19)			2.53(0.14)			2.79(0.16)		

Note: Data presented as mean and Standard Deviation (S.D); One-way ANOVA used to test the correlation between demographic variables and knowledge.

**Table 7:** Behavior and practice of food handlers in Kenyan high school kitchens.

Practice Items	Mean	Std. Dev.
First thing to do upon entering the kitchen is wash my hands.	4.152	1.463
I always wear my tidy uniform prior to beginning work.	4.064	1.652
I wash my hands when I touch the cooked food.	4.598	1.094
I will perform at least one health check every year.	4.652	0.932
I don’t use cooking tools to taste the food.	4.294	1.429
If I have wounds on my hand prior to coming to the kitchen, I will wear gloves.	2.907	1.916
I use different chopping blocks to deal with the food materials.	2.882	1.772
I dispose of any moldy food.	4.750	0.916
If there are cracks on dishes, I will not use them.	4.569	1.166
I completely disinfect the cutter and chopping block after work every day.	4.230	1.283
I clean and dry the facility after work every day.	4.417	1.360
I disinfect the work area regularly	4.108	1.441
When I wash dishes, I use the three-sinks methods.	2.848	1.748
I need to clean the drainage every day.	3.642	1.785
Behavior and practice	4.008	0.439

**Table 8:** Demographics of food handlers in Kenyan high school kitchens in relation to practice.

Demographics Characteristics		Descriptive Statistics		ANOVA	
		Mean	Std. Deviation	F	Sig.
Gender	Female	3.762	0.409	19.886	0.000
	Male	4.078	0.439		
Age	25–35 years	3.896	0.689	18.119	0.000
	36–45 years	4.070	0.488		
	46–55 years	4.278	0.326		
	56–65 years	3.145	0.000		
	Above 65	2.400	0.000		
Education levels	Primary	4.101	0.410	9.543	0.000
	Secondary	3.931	0.436		
	College	4.051	0.214		
	Degree	2.143	0.295		
Experience	Less than one year	3.931	0.295	17.874	0.000
	One	3.521	0.583		
	Two	4.067	0.227		
	Three	3.987	0.487		
	Four	4.141	0.313		

Note: Data presented as Mean and Std. deviation; One-way ANOVA used to test the correlation between demographic variables and practice.

**Table 9:** Multiple regression on the influence of knowledge on practice of food safety and sanitation.

Variables	Unstandardized Coefficients		Standardized Coefficients		
	B	Std. Error	Beta	t	Sig.
(Constant)	−1.047	0.825		−1.269	0.206
Personal health	−0.368	0.088	−0.226	−4.197	0.000
Food contamination	0.753	0.272	0.152	2.768	0.006
Food transmission diseases	0.827	0.088	0.526	9.376	0.000
Model Summary Statistics					
R	0.664				
R Square	0.441				
Adjusted R Square	0.430				
Model Fitness Statistics					
F	39.235				
Sig.	0.000				
a Dependent Variable: behavior and practice					

Note: Multiple regressions; demonstrate correlation on both knowledge and practice.

## Data Availability

The data presented in this study are available on request from the corresponding author. The data are not publicly available as respondents were promised anonymity.
